# Supplementary material for: TWIST1 Upregulation Is a Potential Target for Reversing Resistance to the CDK4/6 Inhibitor in Metastatic Luminal Breast Cancer Cells
Source: Int J Mol Sci. 2023 Nov 14;24(22):16294. doi: 10.3390/ijms242216294 (PMC10671583; doi:10.3390/ijms242216294)
Supplement: Supplementary file 1 [file ijms-24-16294-s001.zip › Figure S3.pdf]

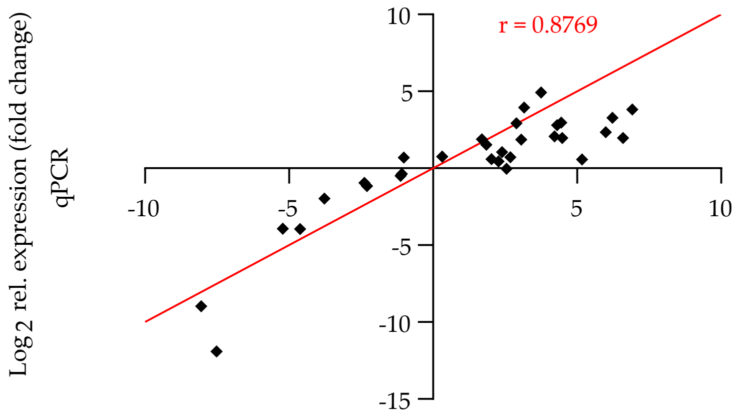

Log 2 rel. expression (fold change)
RNAseq

GENE	Log2 rel.expression (fold change) RNAseq	Log2 rel.expression (fold change) RT-qPCR
CDKN2B	-8.045994697	-8.96578
PYCARD	-7.518215163	-11.9125
NR5A2	-5.221951766	-3.92611
CEACAM5	-4.621880153	-3.9535
CEACAM7	-3.769415513	-1.97177
ABCG2	-2.383293605	-0.94379
RUNX2	-2.297378303	-1.15273
CDKN1B	-1.126575933	-0.49372
KLF4	-1.084799525	-0.35845
ESR1	-1.018363226	0.707862
CDK7	0.313038481	0.765535
CD44	1.69182703	1.887525
PGR	1.852442301	1.521053
FSCN1	2.022967129	0.595609
SNAI2	2.272132493	0.44309
miR210	2.399100381	1.042644
TGFB1	2.551451409	-0.03964
BLHEHE40	2.683283525	0.713414
VIM	2.898929976	2.922306
BCL2	3.063707098	1.858655
DNp63	3.158654884	3.944858
NTRK2	3.754711138	4.923305
MGP	4.221459136	2.074388
PTGS2	4.309265176	2.799447
COL5A1	4.453732184	2.960807
IGFBP3	4.485737984	1.976719
VCAN	5.174700616	0.574395
MMP1	5.993541059	2.334762
miR31	6.22818964	3.277985
SERPINE1	6.596722724	1.971737
TWIST1	6.921966439	3.822044
