# Supplementary material for: TWIST1 Upregulation Is a Potential Target for Reversing Resistance to the CDK4/6 Inhibitor in Metastatic Luminal Breast Cancer Cells
Source: Int J Mol Sci. 2023 Nov 14;24(22):16294. doi: 10.3390/ijms242216294 (PMC10671583; doi:10.3390/ijms242216294)
Supplement: Supplementary file 1 [file ijms-24-16294-s001.zip › Figure S5.pdf]

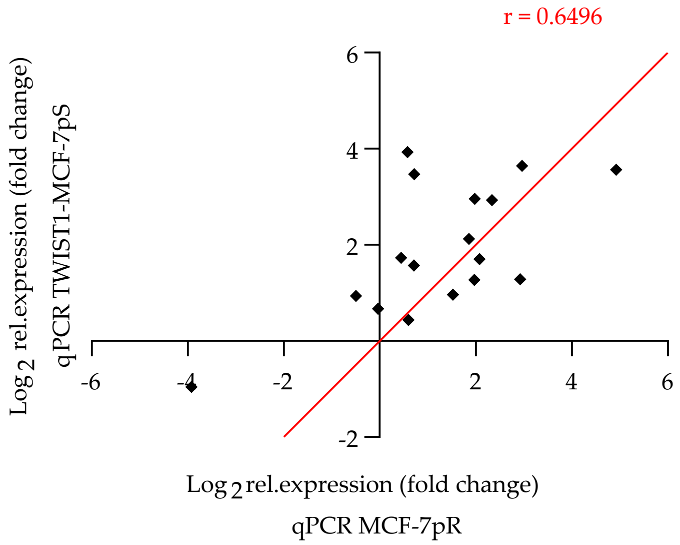

GENE	Log2 rel.expression (fold change) qPCR MCF-7pR	Log2 rel.expression (fold change) qPCR TWIST1-MCF-7pS
NR5A2	-3.92610627101808	-0.953634915895532
CDKN1B	0.707861908025551	1.56984354332004
ESR1	1.52105331415924	0.963309042209582
PGR	0.595609444805636	0.438027435538661
FSCN1	0.443090096076531	1.73029111943991
SNAI2	-0.039639742595887	0.671309737880771
TGFB1	0.713414214994931	3.47278755285724
BLHEHE40	1.8586550671851	2.12632389731824
VIM	4.92330482921388	3.56011125459819
BCL2	2.07438845901685	1.70610681417209
NTRK2	2.96080667540492	3.64446729445308
MGP	1.97671939456297	2.95551600098462
COL5A1	0.574394818767972	3.930469508815
IGFBP3	2.33476205712047	2.93294758293678
VCAN	1.97173702416774	1.26740614097698
MMP1	2.92230562364642	1.28518498959429
SERPINE1	-0.493716832469057	0.938843473605093
