# Supplementary material for: TWIST1 Upregulation Is a Potential Target for Reversing Resistance to the CDK4/6 Inhibitor in Metastatic Luminal Breast Cancer Cells
Source: Int J Mol Sci. 2023 Nov 14;24(22):16294. doi: 10.3390/ijms242216294 (PMC10671583; doi:10.3390/ijms242216294)
Supplement: Supplementary file 1 [file ijms-24-16294-s001.zip › Sup Figures Legends.pdf]

Supplementary Figure Legends

Figure S1. Cell cycle analysis by flow cytometry to examine the effect of palbociclib treatment (1 μM) on MCF-7pS and MCF-7pR cells at 48h (a) and a representative DNA histogram of one experiment (b).

Figure S2. The Scratch Wound Healing Assay showed that cell migration increased in MCF-7pR compared to MCF-7pS after 24 and 48 hours of exposure to a 1 μM treatment compound. Representative images of the assay are shown in panel (a), with the analysis graph depicted in panel (b). The experiment utilized 2-way ANOVA with error bars indicating SEM. The p-values were $^{**}p<0.01$ and $^{***}p<0.001$.

Figure S3. Linear correlation of gene expression as assessed by RNA-Seq (x axis) and RT-qPCR (y axis). r represents the Pearson linear correlation coefficient. The evaluated genes are listed from the lower to the higher $\log_2$ gene expression (fold change) RNA-seq.

Figure S4. CDKN2B silencing in MCF-7pS. Cell sorting plots of Sh-Scramble GFP and Sh-CDKN2B-GFP (a). Western blotting of CDKN2B in sorted cells (b). Dose-response curves in Sh-Scramble and Sh-CDKN2B MCF-7pS cells illustrated the effect of treatment with varying concentrations of palbociclib. Dashed lines denote the IC50 values (c).

Figure S5. Linear Correlation of differentially expressed genes in MCF-7pR being directly bound by TWIST1 within their promoter region.

Figure S6. Normalized proliferation at the combination of both molecules is compared to the expected Bliss score, which was obtained by multiplying the normalized proliferation of single treatments.

Table S1. Top DEG genes derived from RNAseq analysis of MCF-7pR vs MCF-7pS.
