# Supplementary figures and images for: TWIST1 Upregulation Is a Potential Target for Reversing Resistance to the CDK4/6 Inhibitor in Metastatic Luminal Breast Cancer Cells
Source: Int J Mol Sci. 2023 Nov 14;24(22):16294. doi: 10.3390/ijms242216294 (PMC10671583; doi:10.3390/ijms242216294)

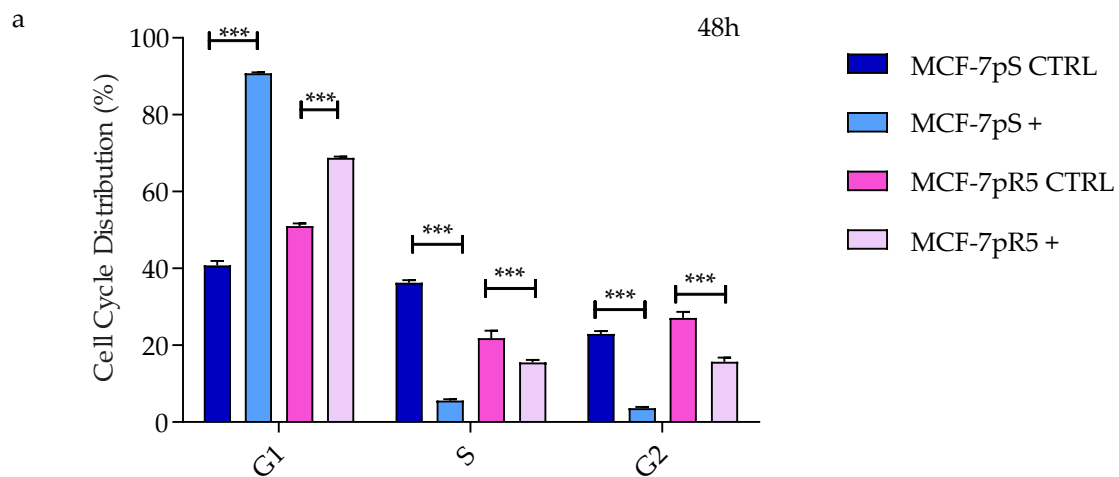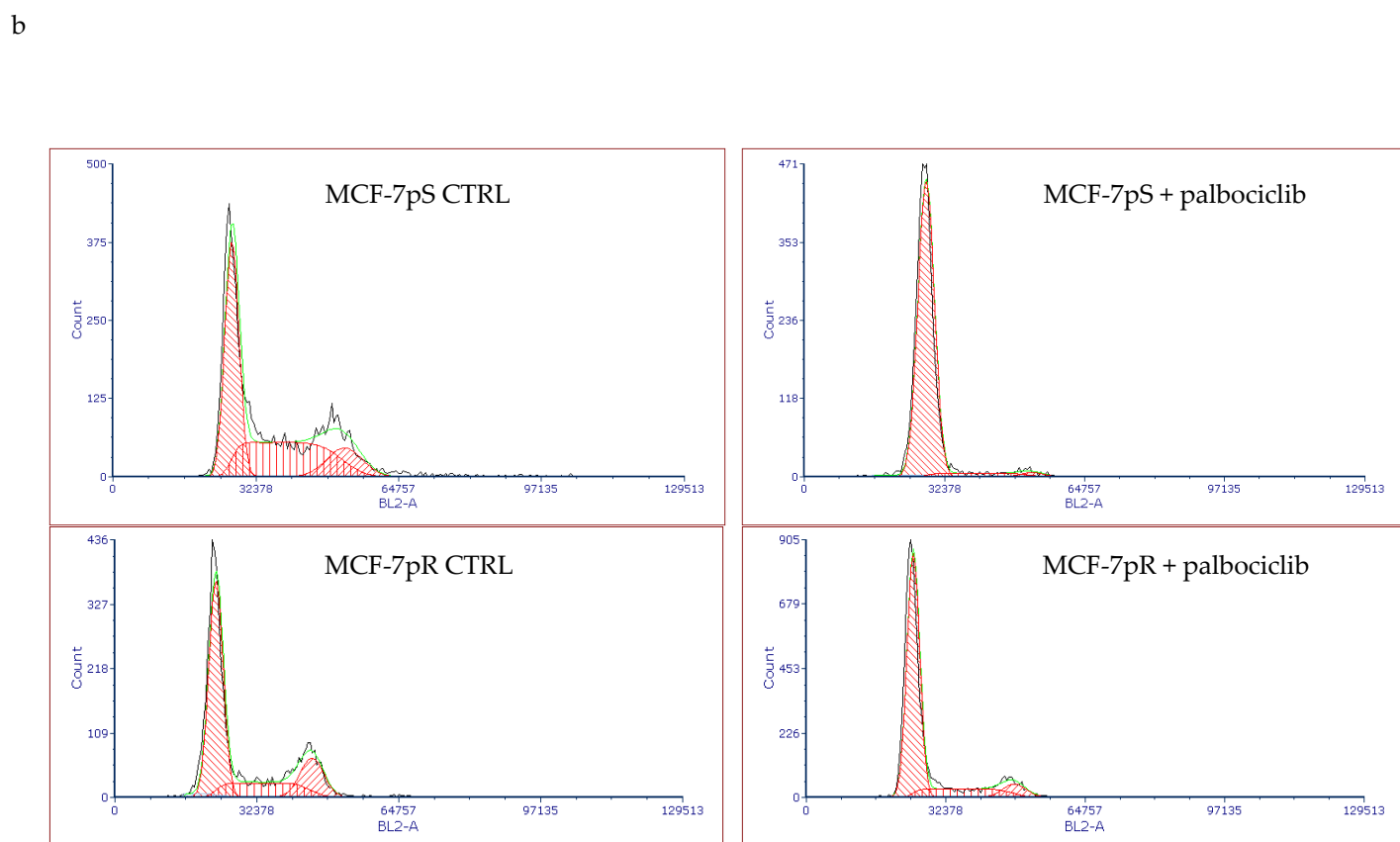

Supplement: Supplementary file 1 [file ijms-24-16294-s001.zip › Figure S1.pdf]

a

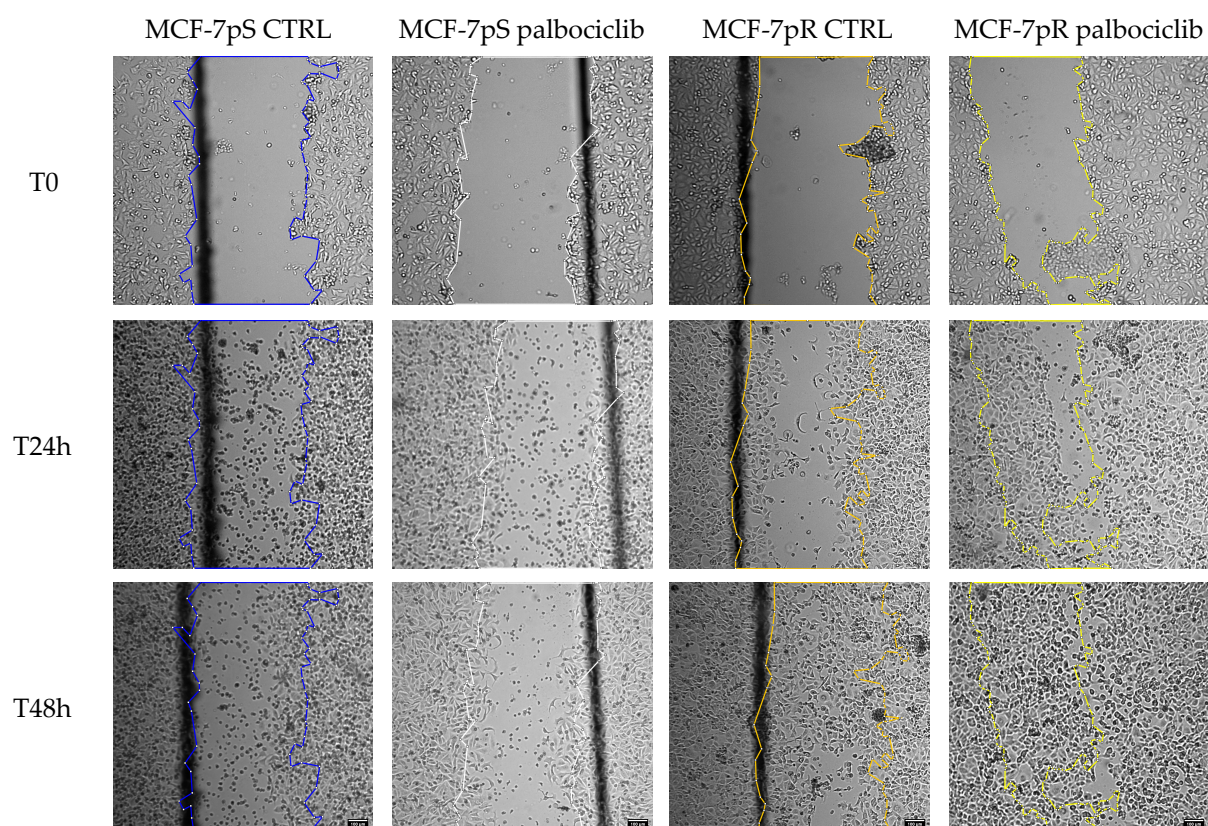

b

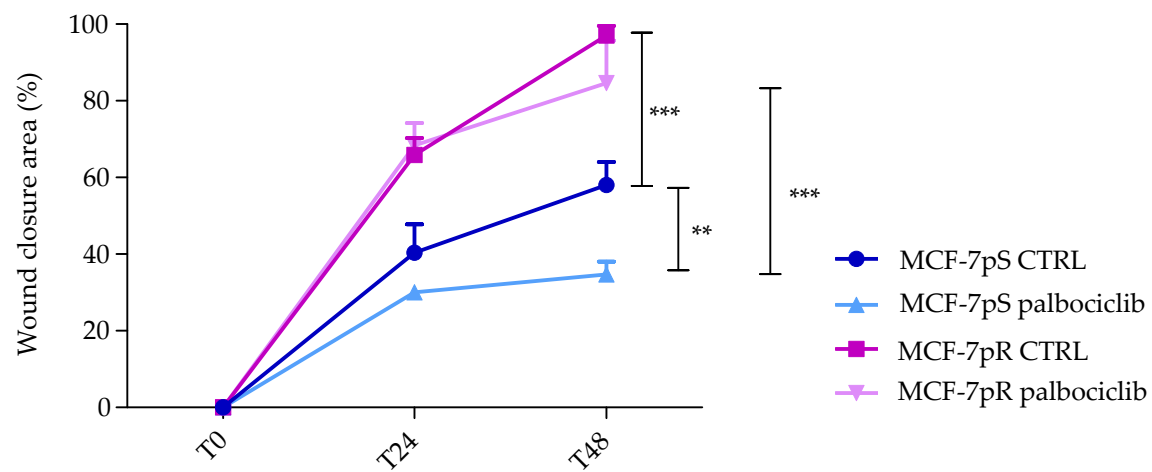

Supplement: Supplementary file 1 [file ijms-24-16294-s001.zip › Figure S2.pdf]

a

Sh Scramble  
GFP+ MCF-7pS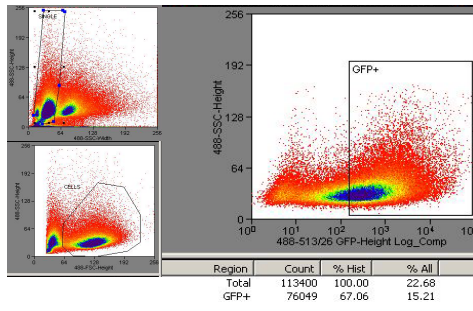Sh CDKN2B  
GFP+ MCF-7pS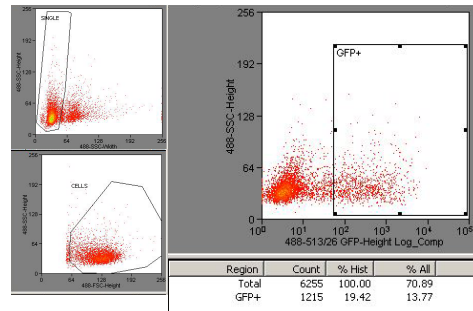

b

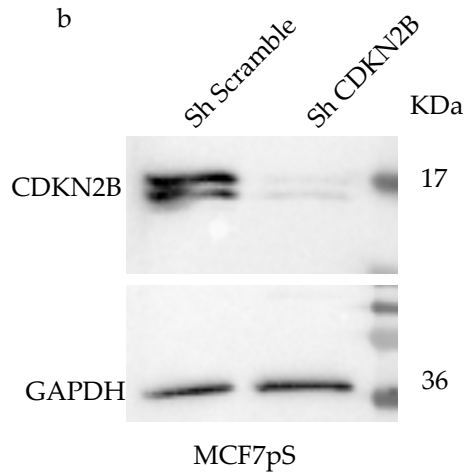

c

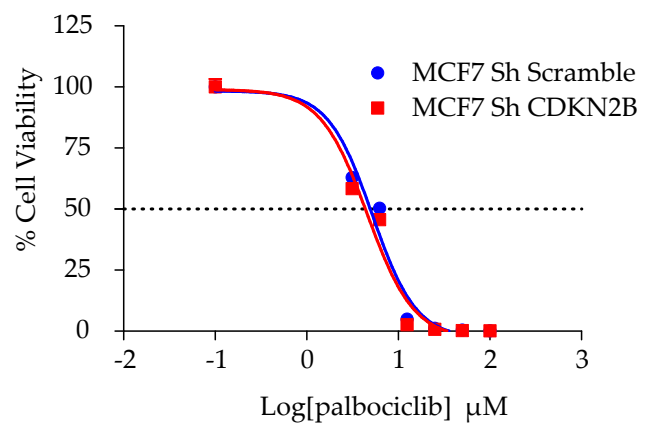

Supplement: Supplementary file 1 [file ijms-24-16294-s001.zip › Figure S4.pdf]

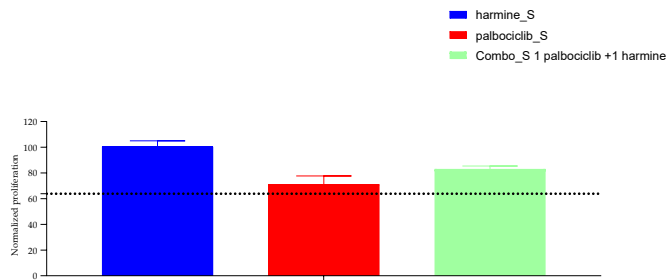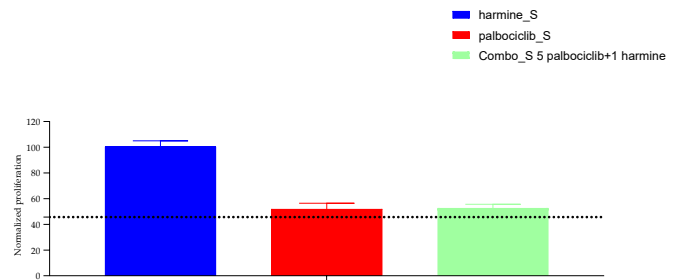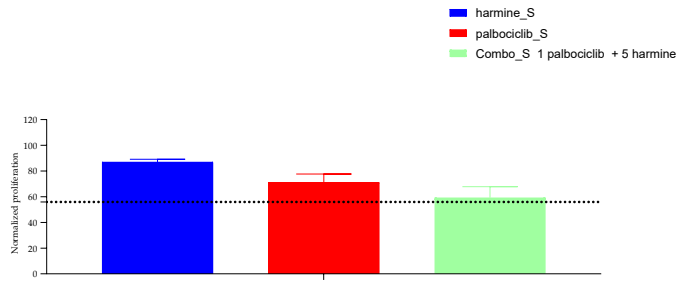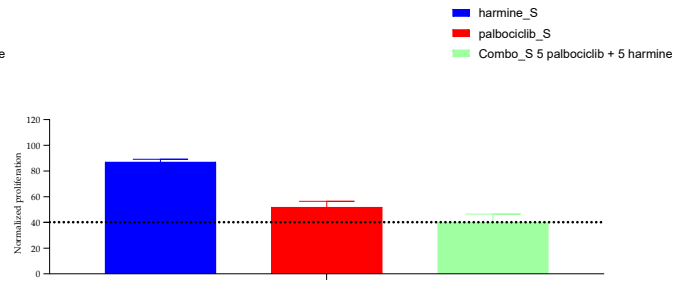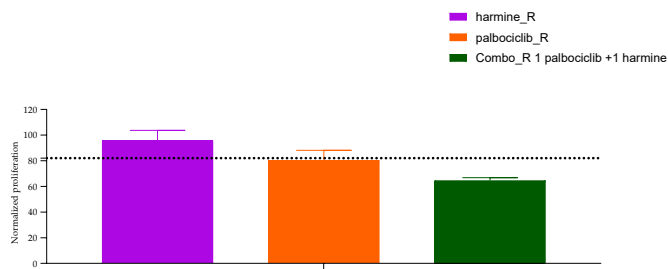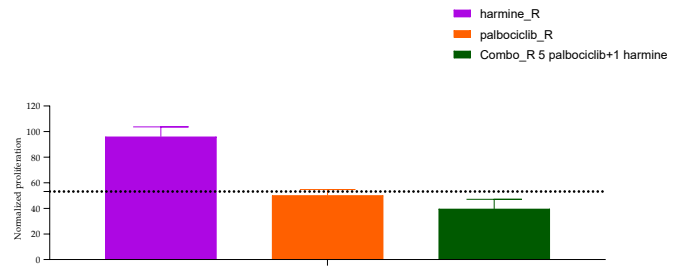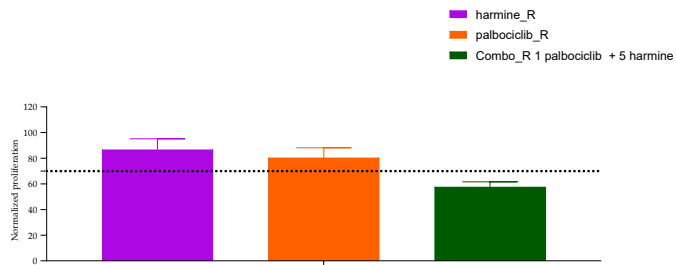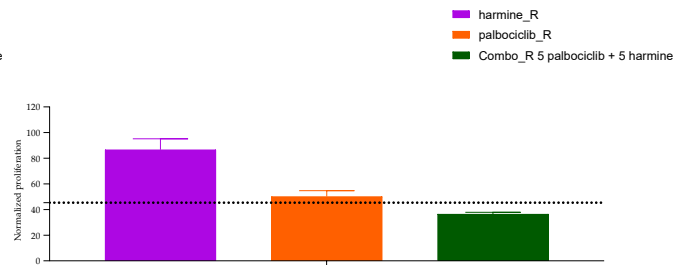

Supplement: Supplementary file 1 [file ijms-24-16294-s001.zip › Figure S6.pdf]
